# Supplementary material for: Discrimination of the Geographical Origin of the Lateral Roots of Aconitum carmichaelii Using the Fingerprint, Multicomponent Quantification, and Chemometric Methods
Source: Molecules. 2019 Nov 14;24(22):4124. doi: 10.3390/molecules24224124 (PMC6891363; doi:10.3390/molecules24224124)
Supplement: Supplementary file 1 [file molecules-24-04124-s001.pdf]

Supplementary Material

Discrimination of the geographical origin of the lateral roots of *Aconitum carmichaelii* using fingerprint, multicomponent quantification, and chemometric methods

Lu-Lin Miao [1,2,3,†], Qin-Mei Zhou [1,2,3,†], Cheng Peng [1,2,*], Chun-Wang Meng [1,2,3], Xiao-Ya Wang [1,2,3] and Liang Xiong [1,2,3,*]

[1] School of Pharmacy, Chengdu University of Traditional Chinese Medicine, Chengdu 611137, China; 18408210835@163.com (L.L.M.); zhqmyx@sina.cn (Q.M.Z.); mengchunw@126.com (C.W.M.); 18234447532@163.com (X.Y.W.)

[2] State Key Laboratory Breeding Base of Systematic Research, Development and Utilization of Chinese Medicine Resources, Chengdu University of Traditional Chinese Medicine, Chengdu 611137, China

[3] Institute of Innovative Medicine Ingredients of Southwest Specialty Medicinal Materials, Chengdu University of Traditional Chinese Medicine, Chengdu 611137, China

* Correspondence: pengchengchengdu@126.com (C.P.); xiling0505@126.com (L.X.)

† These authors contributed equally to this work.

The List of Contents

No	Content	Page
1	Optimization Process of the Extraction Conditions	S1
2	**Table S1.** Six representative alkaloids contents of Fuzi from Jiangyou, Butuo, and Yunnan	S3
3	**Table S2.** Similarity results of HPLC fingerprint of Fuzi from Jiangyou, Butuo, and Yunnan	S4

Optimization Process of the Extraction Conditions.

Study on extraction solvent. Different extraction solvents (dichloromethane, ethyl acetate, methanol, and *v/v* 1:1 mixed solution of isopropanol-ethyl acetate) were used to extract the same batch of Fuzi. All operations were the same except for the extraction solvent. The extract was determined with the chromatographic conditions of Fuzi in Chinese Pharmacopoeia. Comparing the peaks of each group, the extraction rate of isopropanol-ethyl acetate mixed solution (*v/v* 1:1) was higher, and the chromatographic peaks were more abundant. Thus, isopropanol-ethyl acetate mixed solution (*v/v* 1:1) was selected as the extraction solvent in the following study.

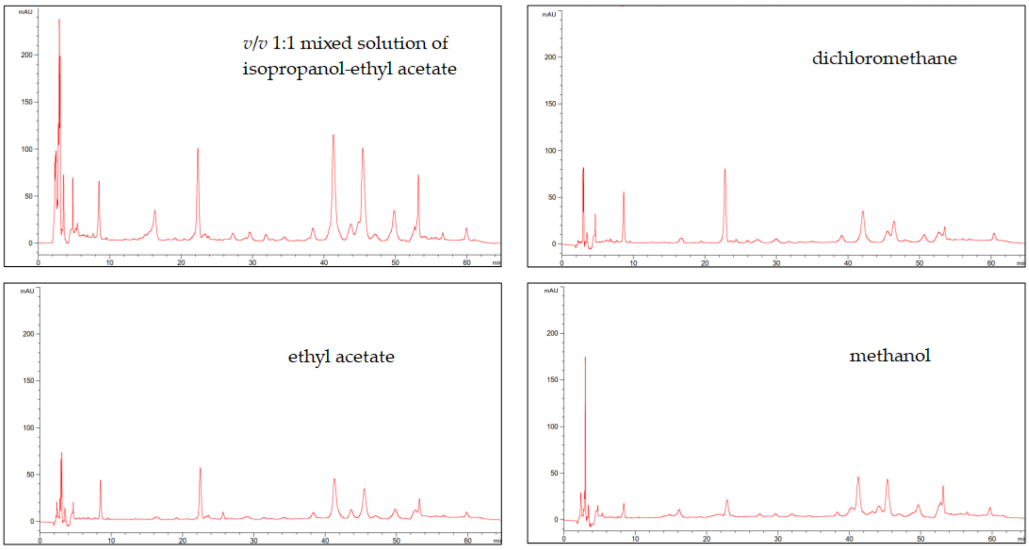

Figure S1. Chromatograms of different extraction solvents.

Study on extraction time. The same batch of Fuzi were extracted in different extraction times (20 min, 30 min, and 40 min). All operations were the same except for the extraction time. Thirty minutes was chosen for ultrasonic extraction according to the chromatographic peak area.

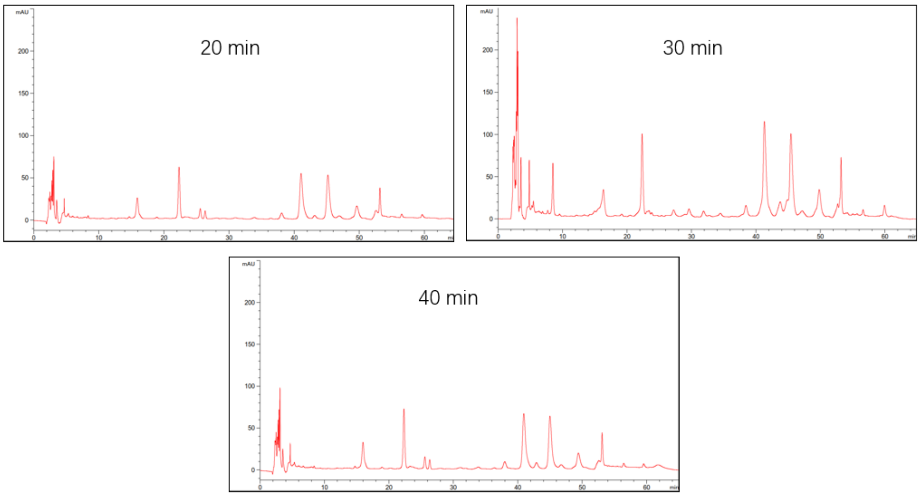

Figure S2. Chromatograms of different extraction time.

Study on ratios of material to liquid. The same batch of Fuzi were extracted with three ratios of material to liquid (1:25, 1:35, and 1:50). All operations were the same except for the

ratio of material to liquid. The extract was determined with the chromatographic conditions of Fuzi in Chinese Pharmacopoeia. There was little difference among three groups, so the ratio of material to liquid (1:25) was selected according to the principle of saving.

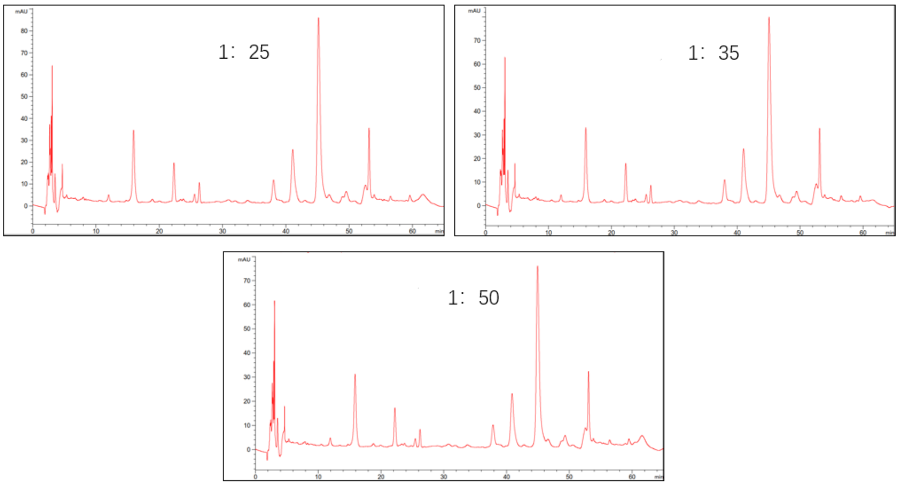

Figure S3. Chromatograms of different ratios of material to liquid.

Table S1. Six representative alkaloids contents of Fuzi from Jiangyou, Butuo, and Yunnan.

Sample	Benzoylmesaconine %	Benzoylaconine %	Benzoylhypaconine %	Mesaconitine %	Hypaconitine %	Aconitine %
JY-1	0.0225	—	0.0059	0.0330	0.0840	0.0037
JY-2	0.0378	0.0037	0.0039	0.0564	0.0524	0.0093
JY-3	0.0295	—	0.0041	0.0422	0.0624	0.0061
JY-4	0.0224	0.0029	0.0069	0.0275	0.0898	0.0062
JY-5	0.0208	—	0.0052	0.0277	0.0782	0.0038
JY-6	0.0280	—	0.0049	0.0373	0.0734	0.0058
JY-7	0.0447	0.0060	0.0051	0.0646	0.0605	0.0219
JY-8	0.0391	0.0034	0.0052	0.0560	0.0673	0.0115
BT-1	0.0202	—	—	0.0940	0.0627	0.0084
BT-2	0.0153	—	—	0.0722	0.0393	0.0097
BT-3	0.0134	—	—	0.0638	0.0632	0.0060
BT-4	0.0062	—	—	0.0379	0.0240	0.0053
BT-5	0.0171	—	—	0.0966	0.0500	0.0141
BT-6	0.0141	—	—	0.0681	0.0263	0.0100
BT-7	0.0306	—	—	0.1612	0.0485	0.0203
BT-8	0.0240	—	—	0.1309	0.0494	0.0136
BT-9	0.0203	—	—	0.0915	0.0521	0.0127
BT-10	0.0262	—	—	0.1383	0.0464	0.0203
BT-11	0.0385	—	—	0.1752	0.0332	0.0236
BT-12	0.0193	—	—	0.1022	0.0560	0.0143
YN-1	0.0236	—	—	0.1344	0.0153	0.0146
YN-2	0.0205	—	—	0.1468	0.0217	0.0163
YN-3	0.0211	—	—	0.1333	0.0237	0.0142
YN-4	0.0253	—	—	0.1728	0.0093	0.0226
YN-5	0.0252	—	—	0.1691	0.0093	0.0221
YN-6	0.0358	—	—	0.2267	0.0108	0.0268
YN-7	0.0178	—	—	0.1619	0.0112	0.0192
YN-8	0.0074	—	—	0.0334	0.0232	0.0017
YN-9	0.0172	—	—	0.1472	0.0106	0.0195
YN-10	0.0276	—	—	0.2021	0.0161	0.0326
YN-11	0.0231	—	—	0.1663	0.0132	0.0263
YN-12	0.0154	—	—	0.0998	0.0086	—

Table S2. Similarity results of HPLC fingerprint of Fuzi from Jiangyou, Butuo, and Yunnan.

	JY-1	JY-2	JY-3	JY-4	JY-5	JY-6	JY-7	JY-8	BT-1	BT-2	BT-3	BT-4	BT-5	BT-6	BT-7	BT-8	BT-9	BT-10	BT-11	BT-12	YN-1	YN-2	YN-3	YN-4	YN-5	YN-6	YN-7	YN-8	YN-9	YN-10	YN-11	YN-12	R
JY-1	1																																
JY-2	0.901	1																															
JY-3	0.947	0.815	1																														
JY-4	0.968	0.845	0.964	1																													
JY-5	0.986	0.904	0.917	0.942	1																												
JY-6	0.949	0.895	0.947	0.935	0.932	1																											
JY-7	0.886	0.984	0.8	0.839	0.892	0.892	1																										

JY-8	0.939	0.985	0.853	0.879	0.947	0.925	0.983	1																									
BT-1	0.847	0.909	0.799	0.812	0.818	0.897	0.924	0.913	1																								
BT-2	0.784	0.905	0.696	0.718	0.767	0.812	0.916	0.895	0.969	1																							
BT-3	0.918	0.934	0.833	0.862	0.901	0.904	0.931	0.946	0.961	0.95	1																						
BT-4	0.794	0.918	0.69	0.709	0.799	0.801	0.917	0.906	0.924	0.972	0.946	1																					
BT-5	0.786	0.9	0.686	0.712	0.771	0.799	0.913	0.899	0.964	0.986	0.951	0.963	1																				
BT-6	0.708	0.881	0.6	0.624	0.702	0.736	0.889	0.861	0.929	0.984	0.906	0.971	0.982	1																			
BT-7	0.664	0.839	0.568	0.594	0.638	0.701	0.856	0.815	0.925	0.971	0.882	0.931	0.973	0.98	1																		

BT-8	0.716	0.862	0.644	0.668	0.682	0.765	0.879	0.843	0.964	0.978	0.907	0.925	0.977	0.972	0.985	1																	
BT-9	0.805	0.92	0.711	0.737	0.785	0.82	0.925	0.909	0.967	0.99	0.963	0.973	0.99	0.978	0.97	0.977	1																
BT-10	0.672	0.829	0.622	0.634	0.638	0.74	0.856	0.809	0.951	0.959	0.873	0.9	0.957	0.954	0.972	0.989	0.953	1															
BT-11	0.555	0.772	0.499	0.51	0.525	0.64	0.799	0.736	0.896	0.926	0.796	0.86	0.923	0.941	0.965	0.97	0.913	0.985	1														
BT-12	0.791	0.907	0.685	0.708	0.781	0.804	0.92	0.906	0.958	0.989	0.952	0.978	0.995	0.986	0.97	0.97	0.992	0.949	0.913	1													
YN-1	0.501	0.73	0.443	0.454	0.476	0.593	0.758	0.693	0.868	0.905	0.761	0.841	0.905	0.927	0.955	0.953	0.89	0.97	0.992	0.892	1												
YN-2	0.535	0.764	0.428	0.45	0.524	0.583	0.781	0.731	0.856	0.926	0.797	0.895	0.937	0.967	0.975	0.951	0.92	0.946	0.966	0.933	0.972	1											
YN-3	0.562	0.777	0.448	0.473	0.555	0.603	0.792	0.751	0.861	0.927	0.809	0.901	0.944	0.969	0.966	0.945	0.92	0.94	0.956	0.939	0.959	0.993	1										

YN-4	0.436	0.685	0.354	0.367	0.407	0.502	0.707	0.641	0.804	0.877	0.722	0.826	0.883	0.92	0.954	0.926	0.872	0.925	0.962	0.874	0.97	0.98	0.96	1									
YN-5	0.442	0.702	0.344	0.363	0.422	0.502	0.722	0.655	0.803	0.885	0.73	0.847	0.891	0.934	0.959	0.925	0.88	0.925	0.962	0.886	0.97	0.989	0.971	0.992	1								
YN-6	0.435	0.702	0.335	0.356	0.418	0.497	0.715	0.65	0.798	0.876	0.726	0.843	0.882	0.926	0.945	0.916	0.875	0.917	0.956	0.875	0.966	0.981	0.966	0.981	0.991	1							
YN-7	0.451	0.694	0.354	0.374	0.429	0.506	0.719	0.655	0.811	0.885	0.734	0.84	0.896	0.931	0.961	0.929	0.879	0.928	0.961	0.889	0.97	0.987	0.97	0.989	0.995	0.982	1						
YN-8	0.752	0.834	0.742	0.738	0.737	0.858	0.853	0.834	0.942	0.911	0.881	0.88	0.889	0.873	0.861	0.903	0.898	0.912	0.864	0.892	0.848	0.811	0.815	0.761	0.764	0.756	0.766	1					
YN-9	0.459	0.716	0.359	0.378	0.447	0.516	0.735	0.672	0.811	0.893	0.745	0.866	0.9	0.942	0.959	0.927	0.889	0.925	0.958	0.897	0.968	0.992	0.974	0.987	0.997	0.992	0.992	0.774	1				
YN-10	0.456	0.698	0.376	0.388	0.43	0.523	0.723	0.659	0.818	0.885	0.736	0.835	0.893	0.926	0.959	0.932	0.881	0.93	0.963	0.884	0.97	0.98	0.961	0.998	0.989	0.978	0.987	0.771	0.985	1			
YN-11	0.466	0.72	0.362	0.382	0.457	0.523	0.746	0.683	0.813	0.895	0.744	0.864	0.904	0.944	0.961	0.926	0.887	0.926	0.958	0.901	0.967	0.992	0.978	0.985	0.995	0.985	0.993	0.776	0.996	0.985	1		

YN-12	0.476	0.736	0.369	0.388	0.473	0.533	0.76	0.697	0.813	0.899	0.749	0.879	0.905	0.951	0.959	0.922	0.89	0.921	0.952	0.906	0.961	0.991	0.978	0.977	0.991	0.98	0.987	0.782	0.994	0.978	0.998	1	
R	0.722	0.885	0.637	0.657	0.703	0.763	0.9	0.866	0.955	0.985	0.914	0.954	0.988	0.99	0.991	0.992	0.985	0.98	0.964	0.985	0.95	0.966	0.963	0.93	0.936	0.929	0.937	0.9	0.942	0.937	0.943	0.944	1
